# Elliptic Curve Cryptography-Based Authentication with Identity Protection for Smart Grids

**DOI:** 10.1371/journal.pone.0151253

**Published:** 2016-03-23

**Authors:** Liping Zhang, Shanyu Tang, He Luo

**Affiliations:** School of Computer Science, China University of Geosciences, Wuhan, Hubei, China; University of South Australia, AUSTRALIA

## Abstract

In a smart grid, the power service provider enables the expected power generation amount to be measured according to current power consumption, thus stabilizing the power system. However, the data transmitted over smart grids are not protected, and then suffer from several types of security threats and attacks. Thus, a robust and efficient authentication protocol should be provided to strength the security of smart grid networks. As the Supervisory Control and Data Acquisition system provides the security protection between the control center and substations in most smart grid environments, we focus on how to secure the communications between the substations and smart appliances. Existing security approaches fail to address the performance-security balance. In this study, we suggest a mitigation authentication protocol based on Elliptic Curve Cryptography with privacy protection by using a tamper-resistant device at the smart appliance side to achieve a delicate balance between performance and security of smart grids. The proposed protocol provides some attractive features such as identity protection, mutual authentication and key agreement. Finally, we demonstrate the completeness of the proposed protocol using the Gong-Needham- Yahalom logic.

## Introduction

Compared with traditional power networks, smart grid networks can avoid excess electricity generation by adjusting the amount of electricity based on the customer’s real-time requirements. In general, the smart grid network can be divided into three levels: control center, substations and smart appliances [1]. In a smart grid network, smart appliances communicate with substations by using smart meters. The smart meters send user’s requirements to the substations, and then the substations transmit the requirements to the control center. Next, according to the received requirements, the control center can allocate adequate power supplies to customers. The Supervisory Control and Data Acquisition system is used to protect the communications between the control center and the substations [2], but the security problems between other two levels remain unsolved. Although the security mechanisms between substations and smart appliances have been researched in recent years, existing security protocols are not robust enough to resist several types of attacks. Therefore, a determined effort should be made to address the security issues associated with the communications between the substations and the smart appliances [3].

As smart meters are used to transmit the real-time electricity demands from customers, the data transmission process could easily suffer from several types of security threats and attacks. To protect the transmitted data, an efficient authentication scheme should be provided. Compared with the authentication protocols designed for other scenarios such as VoIP and Ad Hoc networks, it is more challenging to provide a suitable authentication protocol for smart grids due to its complicated architecture and diverse security requirements. On one hand, the authentication protocols should secure against various types of possible attacks and provide several security features to satisfy the secure requirements of smart grids. For example, the user privacy should be fully considered especially the user’s identity protection, to prevent the adversary from obtaining the information about user’s daily patterns, which may not be important in other application environments. On the other hand, smart grid communications are more sensitive to transmission latency, and so existing security approaches with intensive computation are impractical in smart grid networks.

Recently, several authentication protocols have been proposed [4–19] to protect the data transmission between communication entities. In an attempt to prevent the adversary from obtaining the daily habit of the customer through analyzing the electricity usage pattern, T.W. Chim et al. [4] designed an authentication protocol by using a tamper-resistant device at the smart appliance and a pseudo identity for the smart grid network to protect the privacy of the customer. However the proposed protocol was suffered from impersonation attacks. Since only substations could authenticate smart appliances, the adversary could easily impersonate the substations to cheat the smart appliances. Besides, their protocol failed to provide a key agreement function capable of protecting the communication between substations and smart appliances. Furthermore, since a timestamp was used in the signing module of their protocol, the clock synchronization problem could not be avoided. In order to reduce the computational cost, Mostafa et al. [5] proposed a message authentication mechanism by using the Computational Diffie-Hellman assumption for smart grids. In their protocol, mutual authentication and key agreement were realized by using Diffie-Hellman exchange protocol between the smart meters distributed at different hierarchical networks of the smart grid system, and the subsequent messages could be authenticated by using a shared session key established previously and the hash-based authentication code technique. However, the computational costs of both protocols were still very high due to the usage of expansive exponential operations. In the same year, Qing et al. [6] designed a multicast authentication protocol for smart grids by using one-time signature to reduce the storage cost and the signature size. Because the one-time signature-based multicast authentication could provide short authentication delay and low computation cost, their protocol achieved a good performance. However their work only focused on designing a light-weight authentication protocol, remained the key agreement issue unsolved.

In order to strengthen the security of smart grid communications, Soohyun Oh et al. [7] suggested a mutual authentication and key establishment mechanism based on public key certificates for smart grid. In their protocol, the data concentration unit’s public key certificate and pre-shared long-term key were used to realize the mutual authentication between the data concentration unit and the intelligent devices. But the problem of distributing the shared long-term key limited this protocol’s scalability and applicability. Biometric technique such as fingerprint was also adopted to achieve strong authentication for smart grids [10]. But these protocols are very complex due to the use of biometrics. In 2013, Binod Vaidya et al. proposed an authentication and authorization mechanism for smart grid networks [13]. They realized multi-factor authentication and attribute-based authorization in a smart grid environment by using public key certificates, zero-knowledge and access control technologies. But the heavy computational load could not be avoided since the implement of the public key certificates management and public key cryptography calculation. In the same year, Nicanfar et al. presented a password authenticated group key agreement protocol for smart grid [15]. Although the proposed protocol provided forward and backward secrecy and enhanced the security of communications among the devices, the usage of expansive exponential operations decreased the practical application of the protocol. To reduce the computational cost, a password authenticated key exchange based on Elliptic Curve Cryptography (ECC) was proposed [17]. Compared with previous studies, this protocol was more efficient due to the usage of ECC, but a primitive password should be preloaded between an appliance and the Home Area Network controller, which made this solution hard to scale and might arouse an intractable problem of password table maintenance. Recently, Li et al. proposed fault-diagnosable authentication architecture for advanced metering infrastructure in smart grid [19]. Since this work only focused on authentication, key negotiation was not considered in the proposed authentication mechanism.

According to above analysis, protocol [4] was suffered from impersonate attacks and protocols [5, 19] were vulnerable to eavesdropping since these protocols could not provide key agreement to protect the further communications. Moreover, protocol [17] faced some attacks associated with password. Although some of these protocols achieved good performance, they could not provide security at an acceptable level. Furthermore, other protocols such as [13, 15] were secure against several attacks, but the use of expansive exponential operations, the signature generation, and the verification lead to high computational overhead and communication delay. Therefore, these protocols are not suitable for smart grid. In general, the existing authentication protocols for smart grids mentioned above are insecure against some cryptographic attacks or impractical due to high computational costs. In addition, all the protocols discussed above could not provide privacy protection which is very important in smart grids. Based on these motivations, we proposed a robust and efficient authenticate protocol based on Elliptic Curve Cryptography (ECC) with identity protection for smart grids by using tamper-resistant attractive security properties. As ECC can achieve the same level security with a smaller key size, it offers better performance compared with other public key cryptosystems such as RSA or D-H. Thus, we adopted ECC to realize a mitigation authentication device at the smart appliance without involving time-consuming operations.

Compared with other security approaches, public key cryptosystems can resist most of possible attacks and provide more security properties to achieve a good balance between performance and security. By using ECC, the proposed protocol can achieve the authenticated key agreement with privacy protection at a lower computational cost. Furthermore, according to the characteristics of the smart grid, the control center can be considered fully trustable since it is managed by the government administrators; the substations that have higher computational power are difficult to be compromised than smart appliances; the smart appliances with limited power are more vulnerable to various attacks, and it can be combined with a tamper-resist device to protect the stored information. Taking advantage of above features, in the proposed protocol, a tamper-resist device was used to store secret information to help providing privacy protection through the authentication process. In addition, the control center and the substations can cooperate to complete the initialization process of the authenticated key agreement protocol.

In the proposed protocol, the smart meters are used to transmit the real-time electricity demands from customers intelligently. In order to protect the transmitted data, mutual authentication and a shared key should be provide to protect the further communication between the substation and the smart appliances. In the proposed protocol, the smart meters could control when the authentication protocol begins and which appliances need to be authenticated. Furthermore, the shared key updating could be realized by restarting the authentication process and the smart meter could also control the period of key updating during the communication. Therefore, the smart meter could manage the smart devices intelligently during the authentication process. In this paper, our study focused on the design of the authentication protocol with privacy protection, so the intelligent management of smart meters is beyond the scope of our work.

Burrows-Abadi-Needham (BAN) Logic [20] is the first belief logic widely used to formally analyze the completeness of a cryptographic protocol, but it has some limitations [21]. Gong-Needham-Yahalom (GNY) logic [22] is one of the famous extensions to overcome the inherent limitations of BAN; and it has successfully disclosed redundancies or found defects in several protocols. Today, GNY has been used to demonstrate the completeness of several protocols successfully [23]. Therefore, we used the GNY logic to evaluate the security of the proposed protocol in this study.

The rest of this paper is organized as follows. Section 2 briefly describes the Elliptic Curve Cryptosystem. Our newly designed authentication protocol is detailed in Section 3. In Section 4, the completeness of the proposed protocol is proved through Gong-Needham-Yahalom logic. The performance of the proposed protocol is evaluated in Section 5, and the paper is concluded in Section 6.

## Preliminaries

In this section we briefly introduce the basic concepts of the elliptic curve cryptosystem and the corresponding problems associated with it. We also explain the reason for adopting the elliptic curve cryptography.

ECC has been formally applied to public key cryptosystems since 1986. In an elliptic curve cryptosystem, the elliptic curve equation is defined as the form *E*_*p*_(*a*,*b*): *y*^2^ = *x*^3^ + *ax* + *b*(mod *p*) over a prime finite field *F*_*p*_, where *p*>3, *a*,*b* ∈ *F*_*p*_ and 4*a*^3^ + 27*b*^2^ ≠ 0(mod *p*). Given an integer $t\in F_{p}^{*}$ and a point *P* ∈ *E*_*p*_(*a*,*b*), the scalar multiplication *tP* over *E*_*p*_(*a*,*b*) can be defined as *tP* = *P* + *P* + … + *P*_(t times)_ [24]. And the corresponding problems associated with ECC are shown as follows:

Definition 1. Given two points *P* and *Q* over *E*_*p*_(*a*,*b*), the elliptic curve discrete logarithm problem (ECDLP) is to find an integer $t\in F_{p}^{*}$ such that *Q* = *tP*.
Definition 2. Given three points *P*, *sP* and *tP* over *E*_*p*_(*a*,*b*) for $s,t\in F_{p}^{*}$, the computational Diffie-Hellman problem (CDHP) is to find the point *stP* over *E*_*p*_(*a*,*b*).
Definition 3. Given two points *P* and *Q* = *sP* + *tP* over *E*_*p*_(*a*,*b*) for $s,t\in F_{p}^{*}$, the elliptic curve factorization problem (ECFP) is to find two points *sP* and *tP* over *E*_*p*_(*a*,*b*).

We assume that the three problems above are intractable. That is, there is no polynomial time algorithm that can solve these problems with non-negligible probability.

Next, we explain why we adopted ECC to design the authentication protocol for smart grid networks.

1) More complex: Since ECC can be implemented in different ways rather than a single encryption algorithm; it is more complex than RSA. Moreover, the elliptic curve discrete logarithm problem is more difficult to break than the factorization and discrete logarithm problem. Although many researchers have tried to attack ECC, it is still infeasible to break ECC with existing computational resources. Therefore, the security strength of ECC is much stronger than other public key cryptosystems such as RSA or Diffie-Hellman (D-H).

2) Smaller key size: as shown in Table 1, compared with RSA, ECC offers equivalent security with smaller key sizes which implies lower power, bandwidth, and computational requirements. These advantages are very important when public-key cryptography is implemented for low power environments.

**Table 1:** Comparison of the key length between RSA and ECC on the same security level.

Key length of ECC (bits)	Key length of an RSA (bits)	Key length ratio
160	1024	1:6
256	3072	1:12
384	7680	1:20
512	15,360	1:12

3) Computational efficiency: ECC is much more efficient than RSA and D-H public protocols in terms of computation, since implementing scalar multiplication in software and hardware is much more feasible than performing multiplications or exponentiations in them.

According to above attractive properties of ECC, we chose it to design the proposed robust and efficient authentication protocol for smart grids.

## Our Proposed Authentication Scheme

This section details our newly designed authentication protocol based on elliptic curve cryptography for smart grids. Considering the efficiency, ECC version for El-Gamal has been adopted for asymmetric encryption in the proposed protocol where the cycle group used in El-Gamal is taken from elliptic-curve. For the details, please see [24]. There were two phases in the proposed protocol: initialization phase and authentication phase. The procedure of our protocol is described in detail as follows:

### Initialization phase

In this phase, several security parameters used for authentication and key agreement are calculated by the control center and the substations.

1) First, an elliptic curve equation *E*_*p*_(*a*,*b*): *y*^2^ = *x*^3^ + *ax* + *b*(mod *p*) over a prime finite field *F*_*p*_ is selected by the control center. Here *a*,*b* ∈ *F*_*p*_ and 4*a*^3^ + 27*b*^2^ ≠ 0(mod *p*). Next the control center chooses a base point *P* over *E*_*p*_(*a*, *b*) and writes *P* to the tamper-resistant device of *U*_*i*_ as well as the substations.

2) The control center allocates an identity *ID*_*i*_ for each smart appliance *U*_*i*_ and preloads *ID*_*i*_ into the memory of the corresponding tamper-resistant devices. Then the identity *ID*_*i*_ of smart appliance *U*_*i*_ is written in an *ID* table by the control center. Next, the control center submits the identity table to the substation over a secure channel and assigns an identity *SID*_*j*_ for each substation *S*_*j*_. The substation *S*_*j*_ stores the identity *SID*_*j*_ in its memory securely. Finally, a one-way hash function *h*(∙): {0,1}* → {0,1}^*k*^ is selected by the control center. And the substations as well as the tamper-resistant devices store the hash function in their memories.

3) The substation chooses a random integer $s\in_{R}Z_{p}^{*}$ as a secret key for symmetric encryption/decryption. And then it generates a random integer *sk*<*n* as a private key and computes its corresponding public key *pk* = *skP*, where *n* is the order of the base point *P*. The computed public/private key pair (*pk*, *sk*) is used for asymmetric encryption/decryption. Then the substation calculates *C*_1_ = *E*_*s*_(*ID*_*i*_) and *C*_2_ = *SID*_*j*_*P* for every smart appliance *U*_*i*_. The system key *s* and the public/private key pair (*pk*, *sk*) are kept secret by the substation. Furthermore, the substation writes the public key *pk* and the pair secret (*C*_1_, *C*_2_) into each corresponding tamper-resistant device.

If a new smart appliance *U*_*j*_ wants to incorporate into the smart grid, the control center and the substation should cooperate to complete the initialization of the new appliance. First, the control center allocates a new identity *ID*_*j*_ for *U*_*j*_ and records it in the *ID* table. Then it sends the identity of the new smart appliance to the corresponding substation over a secure channel. Having received the message, the substation records the identity in its *ID* table and then computes a secret (*C*_1_, *C*_2_) for the new smart appliance. Finally, the substation writes the point *P*, the one-way hash function, the identity *ID*_*j*_, the public key *pk* and the pair secret (*C*_1_, *C*_2_) into the tamper-resistant device of *U*_*j*_ to achieve the initialization of the new smart appliance.

### Authentication phase

During the authentication process, the substation and the smart appliance *U*_*i*_ perform the following four steps to realize mutual authentication and key agreement.

1) First, the tamper-resistant device of *U*_*i*_ selects an integer $r_{1}\in_{R}Z_{p}^{*}$ randomly to compute *C*_3_ = *e*_*pk*_(*ID*_*i*_‖*C*_1_‖*r*_1_), where *e*_*pk*_(•) denotes the public key encryption function using the substation *S*_*j*_’s public key *pk* and *C*_1_ = *E*_*s*_(*ID*_*i*_) is a secret stored in the tamper-resistant device of *U*_*i*_. Then, the smart appliance *U*_*i*_ sends *C*_3_ = *e*_*pk*_(*ID*_*i*_‖*C*_1_‖*r*_1_) to the substation *S*_*j*_.

2) In this step, the substation *S*_*j*_ obtains *ID*_*i*_, *C*_1_ and *r*_1_ by decrypting the receiving message *C*_3_ via its private key *sk*. Then, it checks whether *ID*_*i*_ is valid by matching it in the *ID* table. If not valid, the authentication process stops. Otherwise, the substation *S*_*j*_ uses the system key *s* to decrypt *C*_1_ and then gets the *ID*_*i*_. Next, it compares the value of *ID*_*i*_ in *C*_3_ with that of *ID*_*i*_ in decrypted message *C*_1_. If they are not equivalent, the substation terminates the authentication process; otherwise, the substation chooses two random integers $r_{2}\in_{R}Z_{p}^{*}$ and $r_{3}\in_{R}Z_{p}^{*}$ to calculate the shared session key *SK* = *h*(*r*_1_‖*r*_2_) and authentication message $C_{4}=E_{r_{1}}(SID_{j}\|r_{2})$, where $E_{r_{1}}(•)$ denotes the secure symmetric encryption algorithm with the secret key *r*_1_. Finally, the substation *S*_*j*_ submits the message (*C*_4_, *r*_3_) to *U*_*i*_.

Here the random integer *r*_3_ needs not be encrypted because it is used to check the freshness of the message only and is not connected with the final session key in any way. Even if the adversary obtained the random integer *r*_3_, the shared key could not be compromised. Thus, the random integer *r*_3_ is transmitted in plaintext, and this method has been widely used in authentication protocols to check the freshness of the message.

3) After receiving the message (*C*_4_, *r*_3_), the smart appliance *U*_*i*_ adopts *r*_1_ to decrypt *C*_4_ and then obtains *r*_2_ and *SID*_*j*_. Then it calculates *SID*_*j*_*P* and checks whether the following equation holds $C_{2}\overset{?}{=}SID_{j}P$. If the equation holds, the smart appliance *U*_*i*_ calculates the shared session key *SK*' = *h*(*r*_1_‖*r*_2_) and the authentication message *C*_5_ = *h*(*SK*'‖(*r*_3_ + 1)). And then *U*_*i*_ submits the authentication message *C*_5_ to the substation *S*_*j*_. Otherwise, the smart appliance *U*_*i*_ rejects the message and terminates the authentication process.

4) Upon receiving the message *C*_5_, the substation *S*_*j*_ checks whether the value of the received *C*_5_ equals to the value of the computed *h*(*SK*‖(*r*_3_ + 1)). If true, the substation *S*_*j*_ sets *SK* as the shared session key with the smart appliance *U*_*i*_; otherwise, it terminates the authentication process.

In the proposed protocol, if the substation *D*_*n*_ needs to be changed, it submits all the shared keys between itself and corresponding smart appliance to the control center over a secure channel and then deletes the *ID* table and the shared keys from its memory. Next the control center transforms *ID* table including all identities of the smart appliance associated with substation *D*_*n*_ and all the session keys submitted from *D*_*n*_ to the new substation *D*_*l*_ over a secure channel. In addition, the control center also chooses a secure one-way hash function and transforms it to the substation *D*_*l*_. After the substation *D*_*l*_ finishes the initialization procedure, it adopts the corresponding shared key to encrypt the secret information including the pair secret (*C*_1_, *C*_2_), the public key *pk* and the hash function. Then the substation *D*_*l*_ can transmit the secret information securely to the corresponding smart appliance. Consequently, the tamper-resistant devices can update the secret information securely. And the new session key between the new substation *D*_*l*_ and the smart appliance can be achieved by running the proposed key agreement protocol to realize the secure and easy change of the substation.

In the proposed protocol, instead of preloading the shared key, the secret (*C*_1_,*C*_2_) as “material” is stored in the tamper-resistant device of the smart appliance to help realize mutual authentication and key agreement. The session key is constructed by two high-entropy random integers chosen by the substation and the smart appliance freely, and the session key varies in each authentication and key agreement process, that is, the secret (*C*_1_,*C*_2_) is not connected with the final computed session key. Thus, even the secret (*C*_1_, *C*_2_) stored in the tamper-resistant device was compromised, the session key would not be leaked and the adversary could not obtain the information transmitted between the smart appliance and the substation encrypted by the session key. Under this case, if the secret (*C*_1_, *C*_2_) was compromised, the message relayed between the smart appliance and the substation would not be exposed to the adversary. On the contrary, if the shared key was preloaded into a tamper-resistant device, the adversary could launch the capture attack to obtain the shared key, and then could use it to decrypt the message communicated between the smart appliance and the substation. In addition, the solution of preloading the shared key requires the substation storing the shared keys for each smart appliance. Once the substation was compromised by the adversary, all the shared keys would be revealed. Furthermore, the associated problems of shared key updating and maintaining make this security measure hard to scale up.

## Security Analysis

Burrows-Abadi-Needham Logic [20] is the first belief logic which has been widely used to formally analyze the completeness of protocols. A great effort has been put into overcoming its limitations [21]. Gong-Needham-Yahalom (GNY) logic [22] is one of these extensions. And it has successfully disclosed redundancies or found defects in several protocols. Therefore, we adopted the GNY logic to evaluate the security of our proposed protocol.

In this section, some formulae and statements used in the GNY logic are introduced first; then the goals and the assumptions of the proposed protocol are set; finally the GNY logic is adopted to prove that the proposed protocol is valid and practical.

### Formulae and statements

In the GNY logic, a formula is a name used to refer to a bit string, which has a particular value in a run [22]. In order to describe the GNY logic, first let symbols *X* and *Y* range over formulae. Then, some formulae used in our authentication proof are introduced and the complete list of all logical postulates is described in [22].

1. (*X*, *Y*): conjunction of two formulae *X* and *Y*.
2. {*X*}_*K*_ and ${\left\{X\right\}}_{K}^{-1}$: symmetrically encrypt and decrypt *X* with the key *K*.
3. {*X*}_+K_ and {*X*}_-K_: asymmetrically encrypt and decrypt *X* with the public key +*K* and the private key -*K*.
4. *h*(*X*): a one-way function of *X*.
*X: *X* is not originated here.

A basic statement reflects some property of a formula. Let symbols *P* and *Q* be principals. The following are statements used in our authentication proof.

1. *P*◁*X*: *P* is told formula *X*.
2. *P*∍*X*: *P* possesses formula *X*.
3. *P*|∼*X*: *P* once conveyed formula *X*.
4. *P*|≡#(*X*): *P* believes that *X* is fresh.
5. *P*|≡*ϕ*(*X*): *P* believes that *X* is recognizable.
6. $P|\equiv P\overset{S}{↔}Q$: *P* believes that *S* is a suitable secret for *P* and *Q*.
7. *P*|⇒*X*: *P* has jurisdiction over *X*.
8. *P*⊲*X: *P* is told that a formula *X* which did not convey previously in the current run.

### Protocol descriptions and goals

In this subsection, some notations are changed to fit the GNY logic and the proposed protocol are transformed into the form of *P*→*Q*:(*X*). In addition, the server’s private key is denoted as–*K* and the corresponding public key is denoted as +*K*.

1. *U* → *S*: ({*ID*_*i*_‖{*ID*_*i*_}_*s*_‖*r*_1_}_+*K*_)
2. $S\to U:({\left\{SID_{j}\|r_{2}\right\}}_{r_{1}},r_{3})$ *U* → *S*: (*h*(*h*(*r*_1_‖*r*_2_)‖(*r*_3_ + 1)))

Next, our goals which consist of three aspects are described in detail.

(1) Message content authentication

Goal 1: *S* believes the message in the first run is recognizable.

$$S|\equiv \phi {\left\{ID_{i}\|{\left\{ID_{i}\right\}}_{s}\|r_{1}\right\}}_{{}_{+K}}$$

Goal 2: *U* believes the message in the second run is recognizable.

$$U|\equiv \phi ({\left\{SID_{j}\|r_{2}\right\}}_{r_{1}},r_{3})$$

Goal 3: *S* believes the message in the third run is recognizable.

$$S|\equiv \phi (h(h(r_{1}\|r_{2})\|(r_{3}+1)))$$

(2) Message origin authentication

Goal 4: *U* believes *S* conveyed the message in the second run.

$$U|\equiv S|∼{\left\{SID_{j}\|r_{2}\right\}}_{r_{1}}$$

Goal 5: *S* believes *U* conveyed the message in the third run.

$$S|\equiv U|∼h(h(r_{1}\|r_{2})\|\left(r_{3}+1\right))$$

(3) Session key material establishment

Goal 6: *U* believes that *S* believes that *SK* is a secret shared between *U* and *S*.

$$U|\equiv S|\equiv U\overset{SK}{↔}S$$

Goal 7: *U* believes that *SK* is a secret shared between *U* and *S*.

$$U|\equiv U\overset{SK}{↔}S$$

Goal 8: *S* believes that *U* possesses *SK*.

S|≡U∍SK

Goal 9: *S* believes that *U* believes that *SK* is a secret shared between *U* and *S*.

$$S|\equiv U|\equiv U\overset{SK}{↔}S$$

### Assumption list

In this subsection, some assumptions are made as follows:

1. The secret key *s* is generated by *S* in the proposed protocol, so *S* possesses *s*. *S* also possesses the private key–*K* and the public key +*K*.
  S∍s,S∍+K,S∍−K
2. Since *S* keeps the identity table, *S* believes that *ID*_*i*_ is recognizable.
  $$S|\equiv \phi (ID_{i})$$
3. Since *U* stores *C*_2_ = *SID*_*j*_*P* secretly and holds the base point *P*. Then *U* can check the *SID*_*j*_ and believes that *SID*_*j*_ is recognizable.
  $$U|\equiv \phi (SID_{j})$$
4. The random integer *r*_1_ is generated by *U* in the protocol, so *U* possesses *r*_1_ and believes that *r*_1_ is fresh. *U* ∍ *r*_1_,*U*|≡#(*r*_1_)
5. The random integer *r*_1_ is generated by *U* as part of the temporal session key in the current run. So, we assume that *U* believes *r*_1_ is a suitable secret for himself and *S*.
  $$U|\equiv U\overset{r_{1}}{↔}S$$
6. The random integer *r*_2_ and *r*_3_ are generated by *S* in the protocol, so *S* possesses *r*_2_ and *r*_3_, and believes that *r*_3_ is recognizable and *r*_2_ is fresh.
  $$S∍r_{3},\;S|\equiv \phi (r_{3}),\;S∍r_{2},S|\equiv \#(r_{2})$$
7. The *SK* generated by *S* is a temporal session key in the current run. So we assume that *S* believes that *SK* is a suitable secret between itself and *U*.
  $$S|\equiv S\overset{SK}{↔}U$$
8. *U* believes that the server *S* is an authority on generating a suitable session key material *SK* shared between *U* and *S*.
  $$U|\equiv S|\Rightarrow U\overset{SK}{↔}S$$

### Authentication proof using GNY logic

In this subsection, we adopt the GNY logic to analyze our protocol. A complete list of all logical postulates and the index in the list is provided [22], such as (*T*1, *P*1), to show how to achieve the goals.

(1) The first run:

$$\frac{S|\equiv \phi (ID_{i}),S∍s}{S|\equiv \phi {\left\{ID_{i}\right\}}_{s},S|\equiv \phi (ID_{i}\|{\left\{ID_{i}\right\}}_{s}\|r_{1})}$$ (R1, R2)

If *S* believes that *ID*_*i*_ is recognizable and *S* possesses the key *s*, then *S* is entitled to believe that the encryption of *ID*_*i*_ with the key *s* is recognizable and then the formula {*ID*_*i*_‖{*ID*_*i*_}_*s*_‖*r*_1_} is also recognizable.

$$\frac{S|\equiv \phi (ID_{i}\|{\left\{ID_{i}\right\}}_{s}\|r_{1}),S∍+K}{S|\equiv \phi \{ID_{i}\|{\left\{ID_{i}\right\}}_{s}\|r_{1}{\}}_{+K}}$$ (R3)

If *S* believes (*ID*_*i*_‖{*ID*_*i*_}_*s*_‖*r*_1_) is recognizable and *S* possesses a public key +*K*, then it believes that the encryption {*ID*_*i*_‖{*ID*_*i*_}_*s*_‖*r*_1_}_+*K*_ is recognizable. Therefore, in the proposed protocol, the server *S* can recognize the message {*ID*_*i*_‖{*ID*_*i*_}_*s*_‖*r*_1_}_+*K*_ in the first run. (Goal 1)

(2) The second run:

$$\frac{U|\equiv \phi (SID_{j}),U∍r_{1}}{U|\equiv \phi (SID_{j}\|r_{2}),U|\equiv \phi {\left\{SID_{j}\|r_{2}\right\}}_{r_{1}}}$$ (R1, R2)

If *U* believes that *SID*_*j*_ is recognizable, then *U* is entitled to believe that the formula (*SID*_*j*_‖*r*_2_) of which *SID*_*j*_ is a component, is recognizable. Since *U* possesses *r*_1_, it also believes that the encryption ${\left\{SID_{j}\|r_{2}\right\}}_{r_{1}}$ is recognizable.

$$\frac{S|\equiv \phi \{SID_{j}\|r_{2}{\}}_{r_{1}}}{S|\equiv \phi (\{SID_{j}\|r_{2}{\}}_{r_{1}},r_{3})}$$ (R1)

If *S* believes ${\left\{SID_{j}\|r_{2}\right\}}_{r_{1}}$ is recognizable, then it is entitled to believe that $\left({\left\{SID_{j}\|r_{2}\right\}}_{r_{1}},r_{3}\right)$, of which ${\left\{SID_{j}\|r_{2}\right\}}_{r_{1}}$ is a component, is recognizable. So, we can conclude that in the proposed protocol, *U* can recognize the message $(\{SID_{j}\|r_{2}{\}}_{r_{1}},\;r_{3})$ in the second run. (Goal 2)

$$\frac{U⊲*{\left\{SID_{j}\|r_{2}\right\}}_{r_{1}},U∍r_{1},U|\equiv U\overset{r_{1}}{↔}S,U|\equiv \phi (SID_{j}\|r_{2}),U|\equiv \#(r_{1})}{U|\equiv S|∼{\left\{SID_{j}\|r_{2}\right\}}_{r_{1}},U|\equiv S∍r_{1}}$$ (I1)

If the following five conditions hold: 1) *U* receives the formula (*SID*_*j*_‖*r*_2_) encrypted with the key *r*_1_ and marked with a not-originated-here mark; 2) *U* possesses *r*_1_; 3) *U* believes that *r*_1_ is a suitable secret for himself and *S*; 4) *U* believes that the formula (*SID*_*j*_‖*r*_2_) is recognizable; and 5) *U* believes that *r*_1_ is fresh. Then *U* is entitled to believe that 1) *S* once conveyed (*SID*_*j*_‖*r*_2_) encrypted with *r*_1_ and 2) *U* believes that the *S* possesses *r*_1_. (Goal 4)

According to the GNY logic, we assume that *U*|≡*S*|⇒*S*|≡*, that is, *U* believes that *S* is honest and competent, and then we can deduce the following statement:

$$\frac{U|\equiv S|\Rightarrow S|\equiv *,U|\equiv S|∼(\{SID_{j}\|r_{2}{\}}_{r_{1}},r_{3})∼>S|\equiv U\overset{SK}{↔}S),U|\equiv \#({\left\{SID_{j}\|r_{2}\right\}}_{r_{1}},r_{3})}{U|\equiv S|\equiv U\overset{SK}{↔}S}$$ (J2)

If *U* believes that *S* is honest and competent; and *U* receives a message $\left({\left\{SID_{j}\|r_{2}\right\}}_{r_{1}},r_{3})∼>S|\equiv U\overset{SK}{↔}S\right)$, which it believes *S* conveyed, then *U* ought to believe that *S* really believes $U\overset{SK}{↔}S$. Therefore, *U* believes that *S* believes that *SK* is a suitable secret between *U* and *S*. (Goal 6)

$$\frac{U|\equiv S|\Rightarrow U\overset{SK}{↔}S,U|\equiv S|\equiv U\overset{SK}{↔}S}{U|\equiv U\overset{SK}{↔}S}$$ (J1)

If *U* believes that *S* is an authority on the statement $U\overset{SK}{↔}S$ and *S* believe in $U\overset{SK}{↔}S$, then *U* ought to believe in $U\overset{SK}{↔}S$ as well. So, *U* believes that *SK* is a suitable secret between *U* and *S*. (Goal 7)

(3) The third flow:

$$\frac{S⊲{\left\{ID_{i}\|{\left\{ID_{i}\right\}}_{s}\|r_{1}\right\}}_{+K},S∍-K}{S⊲(ID_{i}\|{\left\{ID_{i}\right\}}_{s}\|r_{1}),S⊲r_{1}}$$ (T3, T4)

If *S* is told a formula (*ID*_*i*_‖{*ID*_*i*_}_*s*_‖*r*_1_) encrypted with the public key +*K* and it possesses the corresponding private key–*K*, then it is considered to have been told the decrypted contents of that encrypted formula. And it has also been told *r*_1_ as the formula’s components.

$$\frac{S⊲r_{1},S∍r_{2},S∍r_{3}}{S∍r_{1},S∍(r_{1}\|r_{2}),S∍h(r_{1}\|r_{2}),S∍(r_{3}+1)}$$ (P1, P2, P4)

If *S* is told *r*_1_, it is capable of possessing *r*_1_. And if *S* also possesses *r*_2_, it is capable of possessing (*r*_1_‖*r*_2_) and *h*(*r*_1_‖*r*_2_). For the same reason, if *S* possesses *r*_3_ then it possesses (*r*_3_+1).

$$\frac{S∍h(r_{1}\|r_{2}),S∍(r_{3}+1)}{S∍(h(r_{1}\|r_{2})\|\left(r_{3}+1\right))}$$ (P2)

If *S* possesses *h*(*r*_1_‖*r*_2_) and (*r*_3_+1), then it possesses (*h*(*r*_1_‖*r*_2_)‖(*r*_3_ + 1)) as well.

$$\frac{S|\equiv \phi (r_{3})}{S|\equiv \phi (h(r_{1}\|r_{2})\|\left(r_{3}+1\right))}$$ (R1)

If *S* believes that *r*_3_ is recognizable, then *S* believes that (*r*_3_+1) is recognizable and (*h*(*r*_1_‖*r*_2_)‖(*r*_3_ + 1)), of which (*r*_3_+1) is a component, is also recognizable.

$$\frac{S|\equiv \phi (h(r_{1}\|r_{2})\|\left(r_{3}+1)\right),S∍(h(r_{1}\|r_{2})\|\left(r_{3}+1)\right)}{S|\equiv \phi h(h(r_{1}\|r_{2})\|(r_{3}+1))}$$ (R5)

If *S* believes that (*h*(*r*_1_‖*r*_2_)‖(*r*_3_ + 1)) is recognizable and it also possesses (*h*(*r*_1_‖*r*_2_)‖(*r*_3_ + 1)), and then it is entitled to believe that *h*(*h*(*r*_1_‖*r*_2_)‖(*r*_3_ + 1)) is recognizable. So, we can say that *S* believes that the message *h*(*h*(*r*_1_‖*r*_2_)‖(*r*_3_ + 1)) in the third run is recognizable. (Goal 3)

$$\frac{S|\equiv \#(r_{2}),S∍(r_{1}\|r_{2})}{S|\equiv \#(r_{1}\|r_{2}),S|\equiv \#(h(r_{1}\|r_{2}))}$$ (F1, F10)

If *S* believes *r*_2_ is fresh then it is entitled to believe that *h*(*r*_1_‖*r*_2_) is fresh. And then if *S* also possesses (*r*_1_‖*r*_2_), it is entitled to believe that *h*(*r*_1_‖*r*_2_) is fresh.

$$\frac{S⊲*h((r_{3}+1),<SK>),S∍((r_{3}+1),SK),S|\equiv S\overset{SK}{↔}U,S|\equiv \#(SK)}{S|\equiv U|∼((r_{3}+1),<SK>),S|\equiv U|∼h((r_{3}+1),<SK>)}$$ (I3)

If all of the following conditions hold: 1) *S* receives a formula consisting of a one way function of (*r*_3_+1) and *SK* marked with a not-originated-here mark; 2) *S* possesses (*r*_3_+1) and *SK*; 3) *S* believes *SK* is a suitable secret for itself and *U*; 4) *S* believes that *SK* is fresh. Then *S* is entitled to believe that *U* once conveyed ((*r*_3_+1), *SK*) and *h*(*h*(*r*_1_‖*r*_2_)‖(*r*_3_ + 1)).Therefore, we can say that *S* believes that the message *h*(*h*(*r*_1_‖*r*_2_)‖(*r*_3_ + 1)) in the third run of the proposed protocol is conveyed from the *U*. (Goal 5)

$$\frac{S|\equiv U|∼((r_{3}+1),SK),S|\equiv \#(SK)}{S|\equiv U|∼SK,S|\equiv U∍SK}$$ (I6, I7)

If *S* believes that *U* once conveyed the formula ((*r*_3_+1), *SK*), then it is entitled to believe that *U* once conveyed *SK*. And if *S* also believes that *SK* is fresh, then it is entitled to believe that *U* possesses *SK*. Therefore, *S* believes that *SK* is possessed by *U*. (Goal 8)

According to the GNY logic, we assume that *U*|≡*S*|⇒*S*|≡*, that is, *S* believes that *U* is honest and competent, and then we can deduce the following statement:

$$\frac{S|\equiv U|\Rightarrow U|\equiv *,S|\equiv U|∼(h(SK\|\left(r_{3}+1\right))∼>U|\equiv U\overset{SK}{↔}S),S|\equiv \#(SK\|\left(r_{3}+1\right))}{S|\equiv U|\equiv U\overset{SK}{↔}S}$$ (J2)

If *S* believes that *U* is honest and competent, and *S* receives a message $h(SK\|\left(r_{3}+1)\right)∼>U|\equiv U\overset{SK}{↔}S$ which it believes is conveyed by *U*, then *S* ought to believe that *U* really believes $U\overset{SK}{↔}S$. So, we can conclude that in the proposed protocol, *S* believes that *SK* is a suitable secret between *U* and *S*. (Goal 9)

## Complexity Analysis

In this section, we first summarize the functionalities of the proposed protocol, and then evaluate the computational cost of the protocol.

As an attractive feature, our protocol provides identity protection including the identities of the smart appliance and the substation. In the proposed protocol, the adversary cannot obtain the real identities of the smart appliance and the substation since the identities are transmitted in ciphertext. So even if the adversary compromises the secret (*C*_1_, *C*_2_) stored in the tamper-resistant device and intercepts all the messages transmitted between the smart appliance and the substation, she/he cannot obtain the real identities of the smart appliance and the substation. In addition, the proposed protocol also provides mutual authentication and key agreement to protect the communications between the smart appliance and the substation. Next, we compare the computational cost of the proposed protocol with other related protocols. Some notations are defined as follows:

1. *T*_*m*_: the time for executing a modular exponentiation operation.
2. *T*_*e*_: the time for executing a scalar multiplication operation of elliptic curve.
3. *T*_*h*_: the time for executing a one-way hash function.
4. *T*_*se*_: the time for executing a symmetric key encryption operation.
5. *T*_*sd*_: the time for executing a symmetric key decryption operation.
6. *T*_*ae*_: the time for executing an asymmetric key encryption operation.
7. *T*_*ad*_: the time for executing an asymmetric key decryption operation.
8. *T*_*hmac*_: the time for executing a Hash-based Message Authentication Code (HMAC) operation

As shown in Table 2, in the proposed protocol, the computational cost at the substation *S*_*j*_ side is *T*_*e*_+*T*_*se*_ during the initialization phase. One scalar multiplication operation *T*_*e*_ is used to compute the secret *C*_2_ = *SID*_*j*_*P*. And one symmetric key encryption operation *T*_*se*_ is used to generate another secret *C*_1_ = *E*_*s*_(*ID*_*i*_) through using the system key *s*. In the authentication phase, the computational cost at the substation *S*_*j*_ side is *T*_*ad*_ +*T*_*h*_ +*T*_*sd*_+ *T*_*se*_, and the computational cost at the smart appliance *U*_*i*_ side is *T*_*ae*_ + *T*_*sd*_ + *T*_*e*_ + *T*_*h*_. The smart appliance *U*_*i*_ takes one asymmetric key encryption operation via the substation *S*_*j*_’s public key *pk* to generate *C*_3_ = *e*_*pk*_(*ID*_*i*_‖*C*_1_‖*r*_1_); takes one symmetric key decryption operation to get *SID*_*j*_ and *r*_2_; takes one scalar multiplication operation to compute *SID*_*j*_*P*; and takes a one-way hash function operation to calculate *C*_5_ = *h*(*SK*'‖(*r*_3_ + 1)). The substation *S*_*j*_ takes one asymmetric key decryption operation to get the smart appliance *U*_*i*_’s identity *ID*_*i*_, the random integer *r*_1_ and the authentication message *C*_1_; takes a one-way hash function operation to obtain *h*(*SK*‖(*r*_3_ + 1)); and takes one symmetric key decryption operation and one symmetric key encryption operation. So, the total computational cost of the proposed protocol is 2*T*_*e*_+*T*_*ae*_+*T*_*ad*_ +2*T*_*sd*_+2*T*_*se*_+2*T*_*h*_. The theoretical analysis and experimental results [25] show that the modular exponentiation operation *T*_*m*_ and the asymmetric key encryption/decryption operations *T*_*ae*_/*T*_*ad*_ are much higher than that of the symmetric key encryption/decryption operations *T*_*se*_/*T*_*sd*_ and the scalar multiplication operation of elliptic curve *T*_*e*_. In addition, compared with the asymmetric key encryption/decryption operations *T*_*ae*_/*T*_*ad*_ and the modular exponentiation operation *T*_*m*_, the computational cost of hash function operation *T*_*h*_ could be ignored. Close analysis of the data in Table 2, shows that our proposed protocol is more efficient than and Mostafa et al.’s protocol [5], because it eliminates the expansive modular exponentiation operations and reduces the numbers of asymmetric key encryption/decryption operations. In addition, compared with Chim et al.’s protocol [4], our protocol reduces the computational cost at the smart appliance side. Although Chim et al.’s protocol possesses better performance at the substation side in comparison with the proposed protocol, their protocol cannot support mutual authentication and fails to provide a key agreement.

**Table 2:** Computational costs comparison between our protocol and others.

	Our protocol	Chim et al.’s protocol [4]	Mostafa et al.’s protocol [5]
Smart appliance	*T*_*e*_+*T*_*ae*_+*T*_*sd*_+*T*_*h*_	2*T*_*ae*_+*T*_*hmac*_	——-
Substation	*T*_*e*_ +*T*_*ad*_+2*T*_*se*_+ *T*_*sd*_+*T*_*h*_	*T*_*hmac*_	——-
Control center	——-	2*T*_*ad*_	——-
HAN	——-	——-	2*T*_*m*_+*T*_*ae*_+*T*_*ad*_+*T*_*h*_+*T*_*hmac*_
BAN	——-	——-	2*T*_*m*_+ *T*_*ae*_ +*T*_*ad*_+*T*_*h*_
Total	*T*_*ae*_+*T*_*ad*_+2*T*_*h*_+2*T*_*e*_+2*T*_*se*_+2*T*_*sd*_	2*T*_*ae*_+2*T*_*ad*_+ 2*T*_*hmac*_	2*T*_*ae*_+2*T*_*ad*_+2*T*_*h*_+4*T*_*m*_+*T*_*hmac*_

HAN: home area network; BAN: building area network

Then, we discuss the communication and storage overhead by comparing our proposed protocol with other protocols. Since Mostafa et al.’s protocol do not use tamper-resistant device, we only compared storage overhead with Chim et al.’s protocol at the smart appliance side. In our protocol, the smart appliance needs to store a hash function and the secure information (*C*_1_, *C*_2_, *pk*, *P*), where *C*_1_, *C*_2_, and *P* are 1024 bits, and *pk* is 128 bits. The total storage overhead needed at the tamper-resistant devices in our protocol is 3200 bits. In Chim et al.’s protocol, the tamper-resistant needs to store the public key *Pub*_*cc*_, the secret key *S*_*r*_, a pair private and public key, the identity of smart appliance *RID*_*i*_ and HMAC function. Where *Pub*_*cc*_ is 1024 bits, *S*_*r*_ is 128 bits, *RID*_*i*_ is 32 bits and a pair key is 2048 bits. Therefore, the total overhead at the tamper-resistant devices side in Chim et al.’s protocol is larger than 3232 bits. As shown in Table 3, Compared with Chim et al.’s protocol, our proposed protocol reduced the storage overhead at the tamper-resistant side.

**Table 3:** Communication costs and storage overhead comparison between our protocol and others.

	Our protocol	Chim et al.’s protocol [4]	Mostafa et al.’s protocol [5]
Storage overhead (tamper-resistance devise side)	3200 bits	3232 bits	——-
Communication cost	608 bits	4448 bits	3744 bits

We hereby present the communication overhead of the proposed protocol. In our experiments, the user’s *ID* was 32 bits, the timestamp was 32 bits, the random number was 64 bits, the signature was 160 bits, and a modular exponentiation was 512 bits. In addition, the output of a 256-bit AES was based on the input of the plaintext. We assume that RSA was adopted as public key encryption/decryption algorithm in protocols [4, 5].The communication cost comparisons between our protocol and others are shown in Table 3. In our proposed protocol, the average communication cost was 608 bits. Compared with the protocols in [4, 5], the proposed protocol scaled down the communication cost significantly.

## Conclusion

An efficient authentication protocol with identity protection for smart grids has been proposed in this paper. In the proposed protocol, based on elliptic curve cryptography the substations and smart appliances realized mutual authentication and key agreement via a tamper-resistant device. In addition, the identities of the smart appliance and the substation are transmitted in ciphertext in the proposed protocol. So the adversary cannot obtain the real identities of the smart appliance and the substation. Furthermore, the completeness of the proposed protocol is demonstrated by Gong, Needham, and Yahalom (GNY) logic. And performance analysis shows that our proposed protocol increases efficiency in comparison with other related protocols. Therefore, the proposed protocol is more suitable for the smart grids.
